# Closed posteromedial dislocation of ankle in a 12 year-old boy: a case report

**DOI:** 10.4076/1757-1626-2-6550

**Published:** 2009-07-17

**Authors:** Yüksel Yurttaş, Volkan Kilinçoğlu, Serdar Toker, Mustafa Kürklü, Atil Atilla, Mustafa Başbozkurt

**Affiliations:** 1 Department of Orthopaedics and Traumatology, Gulhane Military School of MedicineGeneral Dr. Tevfik Sağlam Cad.Etlik/AnkaraTurkey; 2 Department of Orthopaedics and Traumatology, Dumlupinar University School of MedicineTavsanli yolu 10.km.43270, KutahyaTurkey; 3 Gulhane Military School of MedicineGeneral Dr. Tevfik Sağlam Cad.Etlik/AnkaraTurkey

## Abstract

Ankle fractures and fracture dislocations are common injuries in orthopaedic practice however pure ankle dislocation without an associated fracture is extremely rare. There are a few cases reporting such a lesion in the literature. Also this injuries are generally open high energy trauma injuries. Closed treatments are reported to be effective and ligament injuries are generally not reported. In this study, we report a closed pure posteromedial ankle dislocation with anterior talofibular ligament rupture and its treatment and outcome in a 12 year-old boy. We think that this is an extremely rare lesion.

## Introduction

Pure dislocation (dislocation without associated fractures) of the posterior border of the tibia is an extremely rare injury [1-5]. The rarity is due to mechanical efficacy of the ankle mortise and resistance of the ankle ligaments being greater than bone making fracture common [6]. Sporadic cases [7-10] and a few series with a limited number of adult patients have been published [11-17]. Among skeletally mature patients, open injuries are reported to be more frequent however only a few patients with posterior dislocation and entrapment of the entire distal part of the fibula behind the tibia without associated fracture has been reported in the pediatric age group [18]. In this case report, we present one of these rare injuries; a closed true posteromedial dislocation of the ankle without associated fracture of the ankle region in a 12 year-old boy and its treatment with closed reduction and below-knee casting.

## Case presentation

**Figure 1.**
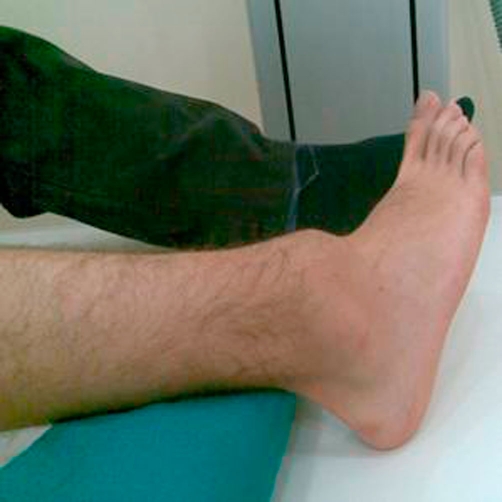
The photograph of the ankle in the emergency room showing posterior dislocation.

**Figure 2.**
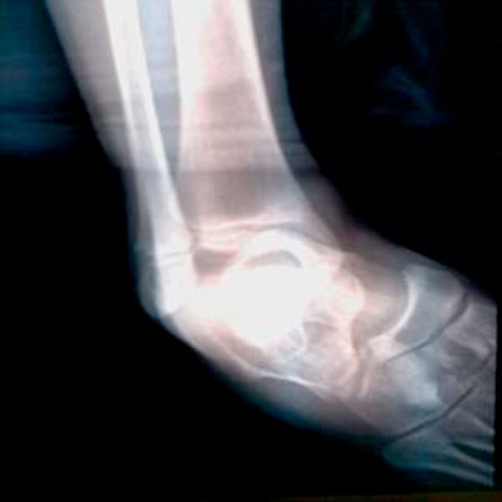
AP X-ray graphy of the ankle representing medial dislocation.

**Figure 3.**
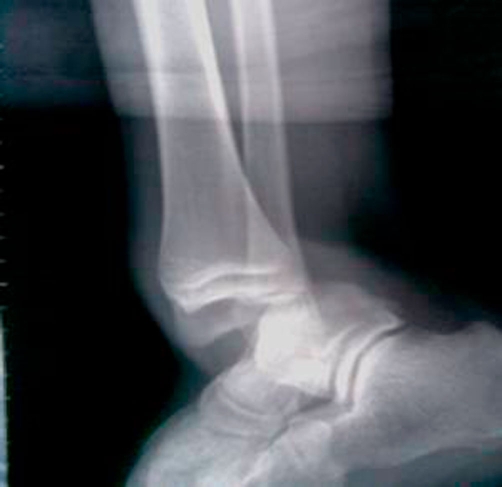
Lateral X-ray ankle showing posterior dislocation.

**Figure 4.**
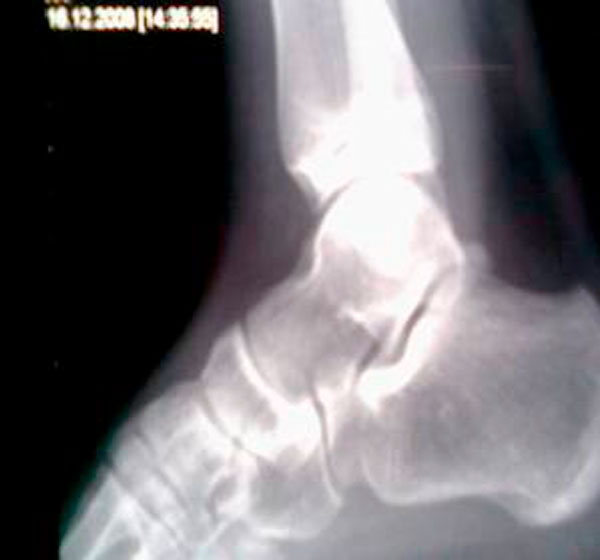
Lateral X-ray of the ankle after reduction.

**Figure 5.**
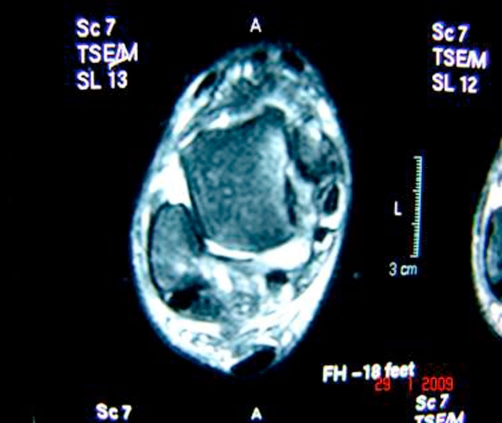
Magnetic resonance imaging showing anterior cruciate ligament rupture.

A 12 year-old Turkish boy from Turkey, referred to emergency room following right ankle injury while running. In initial examination, the ankle swelling and deformity drew attention. The hindfoot seemed to be displaced posterior to the ankle (Figure 1). There were no signs of injury to the neurovascular structures. Ankle X-ray revealed frank posteromedial dislocation of the talus without associated fracture (Figures 2, 3). Following 50 mg diazepam administration intermuscularly, closed reduction was performed by longitudinal traction with one hand on the heel and the other on the forefoot and the limb was placed in a short leg cast. Control X-ray revealed a normal tibio-talar alignment (Figure 4). Magnetic resonance imaging (MRI) revealed an anterior talofibular ligament rupture (Figure 5). The patient was interned, and the extremity was elevated. Following pain relief and decrease in swelling and oedema, the patient was allowed to ambulate without weight-bearing using crutches. Weight-bearing was not allowed for a total of 4 weeks. He was instructed to elevate his leg, take NSAIDS, use ice and crutches in a non-weight bearing manner until further evaluation. Follow-up examination at one-month post injury indicated moderate swelling with good range of motion and a congruent reduction. No instability was detected. At this time, he was advised to begin physical therapy and progressively apply weight over the next few weeks, and was cleared to return to activity seven weeks post-injury.

## Discussion

Ankle fractures and fracture dislocations are common injuries in orthopaedic practice however pure ankle dislocation without an associated fracture is extremely rare [3,5,19]. The rarity is described to be due to mechanical efficacy of the ankle mortise and resistance of the ankle ligaments being greater than bone making fracture common [6]. Moehring et al reported a series of 14 patients with pure ankle dislocation who all were young adults (18-41 years of age) and mostly experienced a high-energy trauma with an open fracture in thirteen of the 14 injuries [2]. Rivera et al also reported a series of three cases with pure ankle dislocation of which one was closed posterior dislocation while two others were open dislocations with one posteromedial dislocation [3]. One can suggest that, pure ankle dislocations are rare injuries but pure, closed ankle dislocations are unique and such injuries mostly need to have high energy trauma, like traffic accident or fall from height [6]. In our case, we report a pure, closed posteromedial ankle dislocation produced by a low-energy trauma so we think that, despite the most common ankle dislocation is reported to be posteromedial dislocation, this case may be one of the unique cases among the others.

The mechanism of pure tibiotalar dislocation is thought to be occurred with maximal plantar flexion followed by inversion. In our case, the mechanism of injury also appeared to be plantarflexion and invertion.

**Figure 6.**
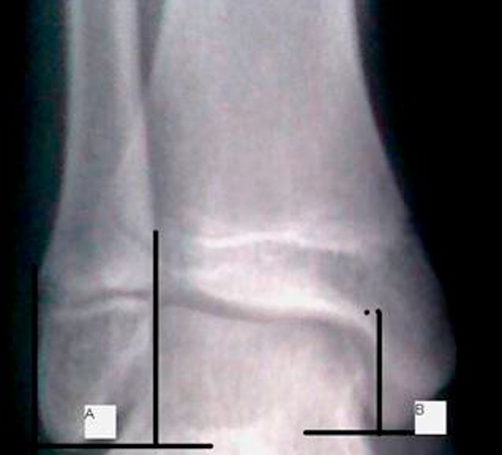
For evaluating the medial malleolus hypoplasia, the ratio between the length of the medial **(B)** and lateral **(A)** malleolus evaluated by the method of Elisé et al. [20] in anteroposterior ankle radiograph and it was found to be in normal ranges (0.62).

Predisposing factors that contribute to the pathogenesis of this lesion are internal malleolus hypoplasia, ligamentous laxity, weakness of the peroneal muscles, and previous ankle sprains [3]. Our patient had neither history of previous ankle sprain nor an evidence of ligamentous laxity or a muscle weakness in the physical examination. ‘For evaluating the medial malleolus hypoplasia, the ratio between the length of the medial (B) and lateral (A) malleolus was evaluated by the method of Elisé et al. [20] in anteroposterior ankle radiograph and it was found to be in normal ranges (eg:0.62) (Figure 6).’

In posteromedial ankle dislocations, associated injuries to the anterolateral neurovascular structures have been reported [1-15,16] however any instability detected after closed reduction in most of the cases [2]. In our case, no neurovascular injury was present but in MRI study, we found anterior talofibular ligament rupture that we could not find a case in the literature reporting this lesion with pure posteromedial ankle dislocation so it may be important to point out that MRI study and clinical reexamination must be performed after reduction of a posteromedial ankle dislocation not only for a neurovascular injury but also for an anterior talofibular ligament lesion.

It is a general agreement that the majority of pure closed ankle dislocations can be treated by closed reduction and cast immobilization. Conservative and operative treatments have been applied to this unusual injury. In closed dislocation, if good reduction is achieved, no operative repairs have been necessary. Wroble et al. stated that closed reduction can be performed easily and optimum treatment is immobilization in a short leg cast for 6 weeks with no weight bearing for the first 3 weeks, following closed reduction [5]. In open dislocation, management consists of immediate reduction followed by debridement and capsular suture and immobilization with a short leg cast [3]. Moehring et al. reported that 12 of 13 patients with open dislocations underwent lateral ligamentous repair [2]. In the present case, we could achieve closed reduction under a mild sedation easily and we applied short leg cast for 4 weeks without weight-bearing. At the end of four weeks, no instability was detected. At this time, he was advised to begin physical therapy and progressively apply weight over the next few weeks, and was cleared to return to activity seven weeks post-injury.

## Conclusions

Closed posteromedial ankle dislocation without an associated fracture is extremely uncommon. Care must be taken for neurovascular injury especially to the anterolateral neurovascular structures but anterior talofibular ligament lesion must also be kept in mind so MRI study, after reduction, can be useful. Closed reduction under sedation is a recommended treatment and prognosis is good. 4-6 weeks of immobilization is generally enough to achieve a stable ankle.

## Abbreviations

AP: Anteroposterior
MRI: magnetic resonance imaging
NSAID: non-steroid antienflamatory drugs
